# Modern treatment approach results in low disease activity in 90% of pregnant rheumatoid arthritis patients: the PreCARA study

**DOI:** 10.1136/annrheumdis-2020-219547

**Published:** 2021-02-10

**Authors:** Hieronymus TW Smeele, Esther Röder, Hetty M Wintjes, Laura JC Kranenburg-van Koppen, Johanna MW Hazes, Radboud JEM Dolhain

**Affiliations:** Rheumatology, Erasmus MC, Rotterdam, The Netherlands

**Keywords:** arthritis, rheumatoid, tumour necrosis factor inhibitors, certolizumab pegol

## Abstract

**Objectives:**

In patients with rheumatoid arthritis (RA), high disease activity impairs fertility outcomes and increases the risk of adverse pregnancy outcomes. The aim of this study was to determine the feasibility of a modern treatment approach, including treat-to-target (T2T) and the prescription of tumour necrosis factor (TNF) inhibitors, in patients with RA with a wish to conceive or who are pregnant.

**Methods:**

Patients were derived from the Preconception Counseling in Active RA (PreCARA) cohort. Patients with a wish to conceive or who are pregnant were treated according to a modified T2T approach, in which the obvious restrictions of pregnancy were taken into account. Results of the PreCARA study were compared with results of the Pregnancy-induced Amelioration of Rheumatoid Arthritis (PARA) study, a historic reference cohort on RA during pregnancy. Patients in the PARA cohort were treated according to the standards of that time (2002–2010). Differences in disease activity over time between the two cohorts were tested using a linear mixed model.

**Results:**

309 patients with RA were included in the PreCARA study, 188 children were born. 47.3% of the patients used a TNF inhibitor at any time during pregnancy. Mean disease activity over time in the PreCARA cohort was lower than in the reference cohort (p<0.001). In the PreCARA cohort, 75.4% of the patients were in low disease activity (LDA) or remission before pregnancy increasing to 90.4% in the third trimester, whereas in the PARA cohort, these percentages were 33.2% and 47.3%, respectively.

**Conclusions:**

This first study on a modern treatment approach in pregnant patients with RA shows that LDA and remission are an attainable goal during pregnancy, with 90.4% of patients achieving this in the third trimester.

**Key messages**

**What is already known about this subject?**

- In patients with rheumatoid arthritis (RA), high disease activity is associated with a prolonged time to pregnancy and is an independent risk factor for lower birth weight of the offspring.
- Tumour necrosis factor (TNF) inhibitors are considered safe during pregnancy; however, it is not known how many patients require treatment with TNF inhibitors during pregnancy.

**What does this study add?**

- In this first study on a modern treatment approach during pregnancy, we showed that low disease activity (LDA) and remission are a feasible goal, with 90.4% of the patients in LDA in the third trimester of pregnancy.

**How might this impact on clinical practice or future developments?**

- In patients with RA with a wish to conceive or who are pregnant, clinicians should strive for remission or LDA.
- The effect of a modern treatment approach on fertility outcomes and pregnancy outcomes should be the focus of further studies.

## Introduction

Rheumatoid arthritis (RA) impairs fertility and pregnancy outcomes.^1^ High disease activity in patients with RA is associated with a prolonged time to pregnancy^2^ and is an independent risk factor for lower birth weight.^3^

Over the last decades, the treatment of RA has evolved: early diagnosis, immediate initiation of disease-modifying antirheumatic drugs (DMARDs), several new approved drugs and a treat-to-target (T2T) approach aiming for remission have resulted in better outcomes for patients.^4–6^ All of these developments are fundamental aspects of both the European League Against Rheumatism (EULAR) and the American College of Rheumatology (ACR) treatment guidelines.^7 8^

Tumour necrosis factor (TNF) inhibitors have revolutionised the treatment in RA. Treatment with TNF inhibitors and/or a combination of DMARDs are considered key elements of a T2T approach.^5 9^ Most TNF inhibitors are considered safe during pregnancy,^10 11^ resulting in the European Medicines Agency (EMA) advising to use certolizumab pegol if clinically needed during pregnancy and adalimumab, etanercept and infliximab if clearly needed during pregnancy.^12^ A drawback of prescribing TNF inhibitors during pregnancy is active transport of these biologics over the placenta into the fetal circulation. This occurs as early as week 18 of gestation.^1^ Therefore, the EULAR points to consider and ACR guidelines conditionally advise to discontinue treatment with most TNF inhibitors before the third trimester of pregnancy. These guidelines advise that certolizumab pegol can be continued throughout pregnancy.^10 13^ To date, the effect of stopping TNF inhibitors during pregnancy on disease activity is not well established, and what treatment strategy should be followed after stopping TNF inhibitors during pregnancy is unknown.

The efficacy of T2T was demonstrated in previous studies; however, whether this approach is feasible in pregnant patients with RA is unknown. The primary aim of our study was to evaluate the feasibility of a modified T2T approach aiming for remission or low disease activity (LDA) in patients with RA with a wish to conceive or who are pregnant. The secondary aims were to determine the percentage of patients that require treatment with TNF inhibitors during pregnancy, and to investigate the effect of stopping TNF inhibitors during pregnancy on disease activity.

## Methods

### Patient population and data collection

Patients were derived from the Preconception Counseling in Active RA (PreCARA) cohort (first inclusion 2011). The PreCARA cohort is an ongoing, prospective cohort study on inflammatory rheumatic diseases and pregnancy. Available data up to 1 October 2020 was used for analysis. The PreCARA study is performed in one tertiary referral hospital (Erasmus MC, Rotterdam) and registered on clinicaltrials.gov with reference number NCT01345071. For the current analysis, patients with RA who delivered and who had at least one visit post partum were used.

Patients were preferably included in the PreCARA study before they got pregnant. Study visits were scheduled every 3 months before conception, during each trimester, and at 6, 12 and 26 weeks post partum. At every visit, patients underwent joint examination, filled in questionnaires, blood was drawn and data on disease activity and frequencies and dosages of conventional synthetic DMARDs (csDMARDs) and biologic DMARDs (bDMARDs) were collected. Information on relevant medical history and previous medication use were collected at inclusion.

### PreCARA treatment protocol

Patients in the PreCARA cohort were treated according to a modified T2T approach aimed at remission. In this protocol, the obvious restrictions of pregnancy, previous response on treatment, previous experienced side effects and patients preference were taken into account. Treatment was, if needed, intensified according to the T2T treatment approach at every study visit. In the PreCARA protocol, first, sulfasalazine and/or hydroxychloroquine were started. Followed by the addition of prednisone (preferably in a maximum daily dosage of 7.5 mg) and/or a TNF inhibitor, preferably certolizumab pegol. Patients were allowed to get pregnant using the TNF inhibitor on which they enrolled in the cohort. TNF inhibitors were stopped during pregnancy at the gestational age as advised by the EULAR,^10^ and a switch to certolizumab pegol or prednisone was considered.

### Data analysis

Disease activity was calculated using the Disease Activity Score with three variables: 28 swollen and tender joint count and C reactive protein (CRP) (DAS28CRP).^14 15^ We stratified disease activity states according to recommendations of the EULAR: remission (DAS28CRP≤2.6), LDA (2.6<DAS28CRP≤3.2), intermediate disease activity (3.2<DAS28CRP≤5.1) and high disease activity (DAS28CRP>5.1).^16^

In line with previous literature, we assessed increase in disease activity between 6 and 12 weeks post partum based on the ‘reversed’ EULAR response criteria.^17^

Results of the PreCARA study were compared with the results of the Pregnancy-induced Amelioration of Rheumatoid Arthritis (PARA) study,^17 18^ a historic reference cohort on RA during pregnancy with a similar study design (inclusion 2002–2010). Patients in the PARA cohort were visited at home and were treated by their own rheumatologist according to the standards of that time for pregnancy, mainly using sulfasalazine, prednisone or no medication. Treatment in this time period was characterised by cautious approach due to insufficient information with regard to breast feeding, gonadotoxic effects and long-term effects in children exposed to immunosuppressive drugs in utero.^19^

### Statistical analysis

Descriptive statistics are presented as numbers (n) and percentages (%). Values are given as mean±SD or median±IQR. We tested categorical data using χ^2^ and Fisher’s exact tests, continuous data using (paired) t-test, analysis of variance and Wilcoxon rank. A two-sided p value of <0.05 was considered significant.

Differences in disease activity over time between the cohorts were tested using linear mixed models with unstructured covariance and random variation within individuals and between individuals. Subgroup analysis for the disease course over time for the use of TNF inhibitors during pregnancy is performed by using linear mixed models with unstructured covariance and random variation within individuals and between individuals. Patients who used a TNF inhibitor at any point during pregnancy were considered TNF inhibitor users during pregnancy. All statistical analyses were performed using Stata V.15 (StataCorp-LP).

### Ethics

This study was approved by the Erasmus MC ethics review board in compliance with Declaration of Helsinki. All patients gave their informed consent.

### Patient and public involvement

Patients were involved in the design of the cohorts. We obtained input from patients in the design of the questionnaires, cohort materials and cohort management. We carefully assessed the burden on participating patients. We intend to share the results to participating patients and will appropriately disseminate the results.

## Results

A total of 587 patients with an inflammatory rheumatic disease, of which 309 women had RA, were included in the PreCARA cohort. 188 children were born (4 twins). A detailed description of the demographics of these women and a description of patients in the PARA cohort are given in table 1.

**Table 1:** Clinical and demographic features of patients with rheumatoid arthritis included in the PreCARA cohort (n=184) and PARA cohort (n=253) that were used for the current data analysis

Variable	PreCARA cohort	PARA cohort	P value
Mean age at delivery, years (SD)	32.8 (3.9)	32.7 (3.8)	0.88
Median disease duration at first visit, years (IQR)	6.8 (3.7–10.7)	4.9 (2.2–9.7)	0.009
Erosive disease, n (%)	52 (28.3)	161 (63.7)	<0.001
Rheumatoid factor positive and/or ACPA positive, n (%)	164 (89.1)	176 (71.8)	<0.001
Nulliparity, n (%)	81 (44.0)	126 (49.8)	0.23
Education level, years of education (SD)	15.9 (3.5)	15.0 (3.0)	0.02
Number of different DMARDs prescribed prior to inclusion in the cohort (IQR)	3 (2–4)	2 (1–3)	<0.001
Number of different csDMARDs prescribed prior to inclusion in the cohort (IQR)	2 (2–3)	2 (1–2)	<0.001
Number of different bDMARDs prescribed prior to inclusion in the cohort (IQR)	1 (0–2)	0 (0–0)	<0.001

ACPA, anti-citrullinated protein antibody; bDMARDs, biologic disease-modifying antirheumatic drugs; csDMARDs, conventional synthetic disease-modifying antirheumatic drugs; DMARDs, disease-modifying antirheumatic drugs.

### Medication use during pregnancy

Table 2 shows the medication used in the PreCARA cohort. Eleven patients (6.0%) did not use any DMARDs during pregnancy. Sulfasalazine, hydroxychloroquine, prednisone and certolizumab pegol were the most commonly used DMARDs. The median daily dosage of prednisone in the third trimester of pregnancy was 5 mg (IQR 5–7.5 mg), 19.0% of the patients used a dosage of >7.5 mg at at least one timepoint during pregnancy. The median daily dosage, for the same period, of hydroxychloroquine was 200 mg (IQR 200–400 mg) and of sulfasalazine was 2000 mg (IQR 1000–2000 mg).

**Table 2:** The percentage of patients in the PreCARA cohort using certain medication during pregnancy (total number of patients=184)

Medication	Last visit before pregnancy n (%) N=116	1st trimester visit n (%) N=167	2nd trimester visit n (%) N=174	3rd trimester visit n (%) N=172	6 weeks postpartum visit n (%) N=170	12 weeks postpartum visit n (%) N=153	26 weeks postpartum visit n (%) N=125
Methotrexate	2 (1.7)	0	0	0	27 (15.9)	34 (22.2)	31 (24.8)
Leflunomide	0	0	0	0	1 (0.6)	2 (1.3)	3 (2.4)
Hydroxychloroquine	77 (66.4)	96 (57.5)	94 (54.0)	93 (54.1)	97 (57.1)	88 (57.5)	70 (56.0)
Sulfasalazine	76 (65.6)	103 (61.7)	104 (59.8)	103 (59.8)	104 (61.2)	95 (62.1)	79 (63.2)
Prednisone	53 (45.7)	69 (41.3)	67 (38.5)	72 (41.9)	67 (39.4)	60 (39.2)	46 (36.8)
Azathioprine	1 (0.9)	3 (1.8)	3 (1.7)	3 (1.7)	1 (0.6)	2 (1.3)	1 (0.8)
Certolizumab pegol	31 (26.7)	38 (22.8)	48 (27.6)	50 (29.1)	47 (27.7)	46 (30.1)	38 (30.4)
Adalimumab	8 (6.9)	8 (4.8)	0	0	5 (2.9)	6 (3.9)	7 (5.6)
Etanercept	19 (16.4)	20 (12.0)	19 (10.9)	6 (3.5)	19 (11.2)	22 (14.4)	16 (12.8)
Infliximab	11 (9.5)	11 (6.6)	4 (2.3)	0	1 (0.6)	2 (1.3)	1 (0.8)
Tocilizumab	2 (1.7)	0	0	0	4 (2.4)	4 (2.6)	6 (4.8)
Golimumab	0	0	0	0	0	0	1 (0.8)
Abatacept	0	0	0	0	1 (0.6)	2 (1.3)	2 (1.6)

### TNF-inhibitor use during pregnancy

Eighty-seven patients (47.3%) used a TNF inhibitor at any time during pregnancy. The most frequently used TNF inhibitor was certolizumab pegol. A total of 26 patients stopped treatment with a TNF inhibitor during pregnancy: adalimumab n=4, infliximab n=7, etanercept n=13, certolizumab pegol n=4. After stopping their TNF, inhibitor, 17 patients (65.4%) used prednisone in the third trimester of pregnancy. Thirteen patients with RA switched their type of TNF inhibitor during pregnancy: switch from adalimumab to certolizumab pegol, n=4; switch from etanercept to certolizumab pegol n=5; switch from infliximab to certolizumab pegol n=4. The median number of weeks of gestation when treatment with infliximab was stopped was 15.3 weeks (IQR 12.7–20.3 weeks), for adalimumab this was 18.4 weeks (IQR 16.9–19.5 weeks), for etanercept 23.4 weeks (IQR 9.9–26.9 weeks) and for certolizumab pegol 35.6 weeks (IQR 26.3–37.4 weeks).

In the third trimester of pregnancy, TNF inhibitors (number of patients that used TNF inhibitors in the third trimester=56) were in 62.5% of the patients combined with sulfasalazine, in 46.4% of the patients with hydroxychloroquine and in 35.7% with prednisone. Twenty-five patients (44.6%) used both sulfasalazine and hydroxychloroquine combined with their TNF inhibitor (with or without prednisone). In patients that did not use a TNF inhibitor (n=116) in the third trimester, sulfasalazine, hydroxychloroquine and prednisone were frequently used in combination. Further, 30.2% of the patients (n=35) used sulfasalazine, hydroxychloroquine and prednisone in combination, while 32.8% of the patients (n=38) used sulfasalazine and hydroxychloroquine without prednisone.

### Medication use in the historic reference cohort

In this cohort of patients, 41.2% did not use any DMARDs during pregnancy (table 3). Patients in the PARA cohort were usually on stable medication: 85% of the patients used the same medication in the first trimester of pregnancy compared with the prepregnancy visit. Prednisone and sulfasalazine were most frequently prescribed during pregnancy. The median daily dosage of prednisone was 7.5 mg (IQR 5–10 mg), 70.6% of the patients used a dosage of >7.5 mg at at least one timepoint during pregnancy. Sulfasalazine was used by 63 (25.7%) patients in the third trimester, in 2 (3.2%) patients sulfasalazine was combined with hydroxychloroquine and in 23 (36.5%) patients with prednisone.

**Table 3:** The percentage of patients in the PARA cohort, a historic reference cohort (2002–2010), using certain medication during pregnancy (total number of patients=253)

Medication	Before pregnancy visit n (%) N=124	1st trimester visit n (%) N=213	2nd trimester visit n (%) N=232	3rd trimester visit n (%) N=245	6 weeks postpartum visit n (%) N=239	12 weeks postpartum visit n (%) N=240	26 weeks postpartum visit n (%) N=222
Methotrexate	0	0	0	0	45 (18.8)	73 (30.4)	89 (40.0)
Leflunomide	0	0	0	0	0	3 (1.3)	4 (1.8)
Hydroxychloroquine	8 (6.5)	5 (2.3)	5 (2.2)	4 (1.6)	10 (4.2)	19 (7.9)	18 (8.1)
Sulfasalazine	42 (33.9)	61 (28.6)	65 (28.0)	63 (25.7)	64 (26.8)	73 (30.4)	70 (31.5)
Prednisone	52 (41.9)	80 (37.6)	87 (37.5)	87 (35.5)	85 (35.6)	89 (37.1)	78 (35.1)
Azathioprine	2 (1.6)	1 (0.5)	1 (0.4)	1 (0.4)	3 (1.3)	2 (0.8)	2 (0.9)
Adalimumab	0	0	0	0	5 (2.1)	7 (2.9)	12 (5.4)
Infliximab	0	0	0	0	1 (0.4)	2 (0.8)	3 (1.4)
Etanercept	0	0	0	0	7 (2.9)	14 (5.8)	13 (5.9)

The medication that is not listed in this table was not prescribed during this study period.

### Disease activity during pregnancy

**Figure 1:**
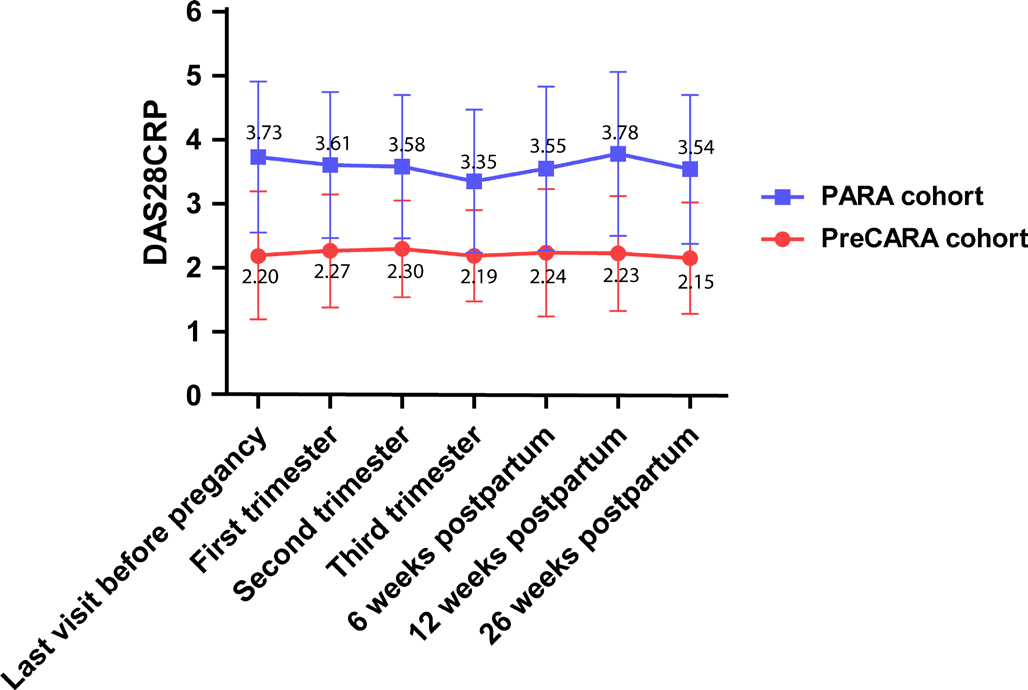
DAS28CRP (mean, SD) scores over time for the PreCARA cohort (modern treatment approach cohort) and the PARA cohort (historic reference cohort). The x‐axis displays specific timepoints before, during and after pregnancy, and the y‐axis represents mean (SD) disease activity. Mean disease activity over time in the PreCARA cohort was lower than in the reference cohort (p<0.001).

Disease activity did not change during pregnancy and postpartum in the PreCARA-cohort (figure 1).

Mean DAS28CRP before pregnancy in the historic reference cohort was 3.73 (SD 1.18) and decreased during pregnancy to DAS28CRP 3.35 (SD 1.12) in the third trimester. Disease activity increased in the postpartum period, the highest observed DAS28CRP 3.78 was at 12 weeks post partum (SD 1.28) (figure 1).

Disease activity over time in the PreCARA cohort was statistically significantly lower than in the historic reference cohort (p<0.001). Also, mean disease activity at every different timepoint in the PreCARA cohort was statistically significant lower (p<0.001).

**Figure 2:**
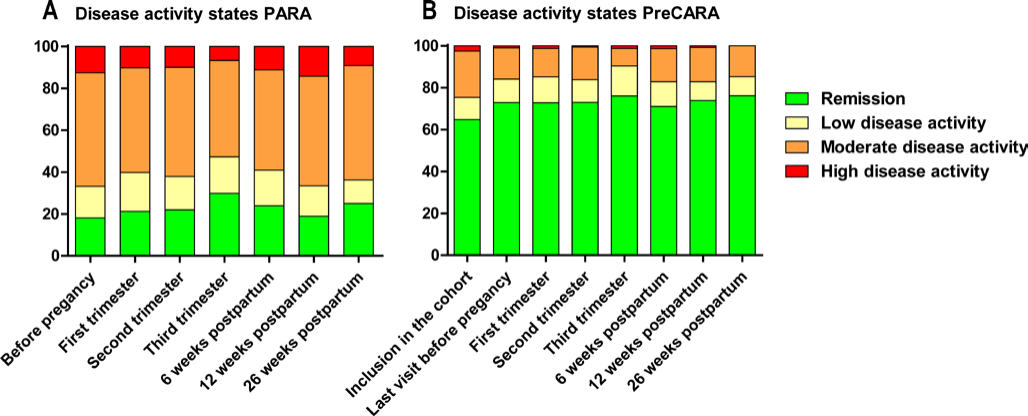
Bar charts showing disease activity states DAS28CRP scores for the PARA cohort (historic reference cohort) (A) and the PreCARA cohort (modern treatment approach cohort) (B). The x‐axis displays the specific timepoints before, during and after pregnancy, and the y‐axis shows the percentage of patients in the different disease activity states. The percentage of patients in moderate or high disease activity was higher at all timepoints in the historic reference cohort compared with the modern treatment approach cohort (p<0.001).

The percentage of patients in remission or LDA in the PreCARA cohort was significantly higher at all timepoints during follow-up compared with the PARA cohort (p<0.001) (figure 2). In the PreCARA cohort, the total number of patients in remission and LDA increased from 64.8% and 75.4% at inclusion to 76.1% and 90.4%, respectively, in the third trimester. The number of patients in remission remained stable post partum. The percentage of patients in different disease activity states was different between the PreCARA cohort and the PARA cohort at all timepoints (p<0.001) (figure 2).

### TNF-inhibitor use and disease activity during pregnancy

Stratified analysis showed no statistically significant difference in disease activity in the third trimester of pregnancy between patients that switched their TNF inhibitor to certolizumab pegol during pregnancy (n=13, DAS28CRP 2.17 (SD 0.73)) versus patients that stopped their TNF inhibitor and used prednisone (n=17, DAS28CRP 2.63 (SD 0.69)) versus patients that used certolizumab pegol throughout pregnancy (n=30, DAS28CRP 2.18 (SD 0.63)), versus patients that stopped their TNF inhibitor and did not use certolizumab pegol nor prednisone (n=8, DAS28CRP 2.23 (SD 0.67)), p=0.13.

Disease activity over time did not differ between patients who used a TNF inhibitor during pregnancy (any use during pregnancy) and patients who did not (p=0.14) (online supplemental figure 1).

10.1136/annrheumdis-2020-219547.supp1 Supplementary data

### Disease activity increase post partum

Not one patient in the PreCARA cohort experienced a severe increase in disease activity post partum, 12.2% of the patients in this cohort experienced a moderate increase. These rates were 5.7% (vs PreCARA cohort, p=0.01) and 21.0%(vs PreCARA cohort, p=0.18) in the PARA cohort, respectively.

## Discussion

Until recently, rheumatologists assumed that almost all patients with RA reach a state of remission during pregnancy independent of treatment; however, more literature shows that over half of the patients still has active disease during pregnancy.^1 17 20^ This highlighted the need for improved care. Our study was the first to evaluate a T2T approach, with the use of TNF inhibitors, low dose prednisone and a combination of DMARDs, in patients with RA with a wish to conceive or get pregnant. Our results show that entering pregnancy in LDA or remission, as advised by ACR guidelines, is attainable when applying T2T. Over 80% of the patients in our study was in LDA at their last visit before pregnancy. Moreover, we showed that applying a T2T approach results in LDA during pregnancy and post partum in a vast majority of patients with RA.

Half of the patients in our study were able to get in LDA or remission using only csDMARDs or prednisone. In a large percentage of the patients, csDMARDs were prescribed in combination. The percentage of patients on prednisone during pregnancy was comparable between our modern treatment approach cohort and the historic reference cohort. However, the dosage of prednisone that was used during pregnancy was considerably lower in the modern treatment approach cohort. In this cohort, it was chosen to preferably prescribe a maximum dosage of 7.5 mg to limit the risk of fertility problems, premature birth, gestational diabetes and high blood pressure since higher doses of prednisone are associated with these complications during pregnancy.^1 21^

We showed that TNF inhibitors were efficacious during pregnancy, no significant difference in disease activity over time between patients that used a TNF inhibitor during pregnancy and patients who used csDMARDs were observed. Patients that were included in our cohort were allowed to get pregnant using their own TNF inhibitor in order to prevent an increase in disease activity by switching therapy. We did, however, observe a larger percentage of patients using certolizumab pegol at inclusion in our cohort (21.2%) than one can expect from the usual Dutch RA patient population.^22^ This could be caused by a switch to certolizumab pegol already before referral to our specialised clinic, since literature shows no to minimal placental transfer of certolizumab pegol during pregnancy.^23^ During pregnancy, TNF inhibitors were stopped at the gestational age advised by the EULAR. Due to reports on high bioavailability of infliximab during pregnancy, it was later chosen to stop infliximab preferably before week 16 of gestation in line with British Society of Rheumatology guidelines.^24^ After stopping a TNF inhibitor during pregnancy, a switch to certolizumab pegol or prednisone was considered to prevent a possible increase in disease activity. Based on expert opinion, certolizumab pegol was arbitrarily stopped at 38 weeks of gestational age in order to minimise maternal infectious complications during delivery. This expert opinion was formed based on guidelines to withhold treatment with a TNF inhibitor before surgery.^25^ TNF inhibitors could be restarted 1 week after a vaginal delivery and 2 weeks after a caesarean section. After delivery, there was no specific preference for one certain TNF inhibitor. However, patients got counselling on breast feeding and many preferred certolizumab pegol due to its robust pharmacokinetic data for use during breast feeding.^26^ According to guidelines, no woman that breast fed used methotrexate. Children that were exposed to TNF inhibitors in utero were vaccinated in line with the Dutch national vaccination policy, in which the first live inactivated vaccine is administered at 14 months. No exceptions for any of the TNF inhibitors were made.

We observed, based on a low number of observations, no statistically significant difference in disease activity between patients that switched TNF-inhibitor treatment during pregnancy and patients that stopped TNF-inhibitor treatment all together. However, this observation is confounded by indication: TNF inhibitors were stopped only in those patients in complete remission after careful consideration of the treating physician and in consultation with the patient. Although TNF inhibitors were stopped, many patients used other medication like prednisone. These results show that physicians are able to distinguish between those patients that have calm disease during pregnancy in which TNF inhibitors can be stopped and patients that, despite having LDA, do require a switch in medication during pregnancy to prevent an increase in disease activity. Our results should not be interpreted as if TNF inhibitors can be stopped during pregnancy without an increased risk of increase in disease activity.

Patients with RA have an increased risk of a flare in disease activity after delivery,^1 20^ not one patient in our modern treatment approach cohort experienced a severe increase in disease activity post partum. In the absence of well-defined criteria, we used criteria based on the ‘reversed’ EULAR response criteria. However, we should note that RA flares are complex, and comprehend more than an increase in disease activity as measured by a physician.^27^

Also, mean disease activity post partum was not different from mean disease activity during pregnancy. This indicates that applying T2T and an immediate restart of medication after delivery may help to prevent an increase in disease activity post partum.

The PreCARA study was designed as an observational study reflecting daily clinical practice in a specialised centre for arthritis and systemic autoimmune disorders and pregnancy. The selection of patients was therefore different from randomised controlled trials (RCTs), like the TICORA trial,^6^ on which the most evidence on T2T is based. Comparing our results with the results of these RCTs might not be appropriate. In previously published studies on T2T in daily clinical practice, the percentage of patients in remission after an extensive follow-up period varies between 52% and 62.6%.^28 29^ The percentage of patients in remission in our study increased from 62.8% at inclusion in the cohort to 74.4% in the third trimester of pregnancy. And although our study cannot be compared directly to these studies, it underscores that in pregnant patients with RA, a T2T approach is feasible too.

Some limitations of our study need to be considered. We compared the results of our modern treatment approach cohort with the results of a historic cohort. Patient characteristics in this historic cohort are slightly different compared with the current patient population. Second, our study could have suffered from selection bias. Our study was performed in one tertiary referral centre, which could have resulted in an over-representation of patients with more severe disease. The significant difference in percentage of patients that had RF or anti-citrullinated protein antibody (ACPA) antibodies in the PreCARA cohort could indicate that this type of bias has occurred. Yet, we showed that even in these patients with more severe disease, LDA during pregnancy is attainable. Furthermore, based on the nature of our study, it was impossible to show that either T2T or new targeted therapies such as TNF inhibitors or combination therapy, or all were responsible for the improved disease outcomes during pregnancy.

We presented in our study only those patients who got pregnant. It is reasonable to speculate that there is an over-representation of patients in LDA or remission in the current study, since active disease is associated with a longer time to pregnancy.^2^ However, for those patients that did not get pregnant, the mean DAS28CRP of all visits during their wish to conceive was 2.36 (SD 1.00). Therefore, we conclude that selection bias based on disease activity is not a relevant factor in our study.

Our study has several strengths. This is the first study to prospectively collect results of a T2T approach in a large cohort of pregnant patients with RA. And, the study was performed in only one tertiary referral hospital, which limited the variation on management of the disease between healthcare professionals. Moreover, the results of the current cohort will allow us to study the effect of T2T and TNF inhibitors on fertility outcomes and pregnancy outcomes in patients with RA in future studies.

The findings of our study should be applied in daily clinical practice. We advise clinicians to apply a T2T approach, including prescribing TNF inhibitors, in all patients with a wish to conceive and during pregnancy. We showed that patients can get pregnant with the TNF inhibitor they already used before pregnancy, and TNF inhibitors can be switched during pregnancy, without an increase in disease activity. Moreover, we advise pregnancy counselling and regular visits during pregnancy and post partum like performed in our specialised hospital. This extra care will contribute to the improved disease outcomes like we observed in our study.

In conclusion, we showed that a modern treatment approach results in LDA or remission in 90% of pregnant patients with RA. Therefore, LDA or remission should also be strived for in this group of patients, despite the obvious restrictions on medication use during pregnancy.

## Data Availability

No data are available.
