# Supplementary material for: Modern treatment approach results in low disease activity in 90% of pregnant rheumatoid arthritis patients: the PreCARA study
Source: Ann Rheum Dis. 2021 Feb 10;80(7):859–64. doi: 10.1136/annrheumdis-2020-219547 (PMC8237196; doi:10.1136/annrheumdis-2020-219547)
Supplement: Supplementary data [file annrheumdis-2020-219547supp001.pdf]

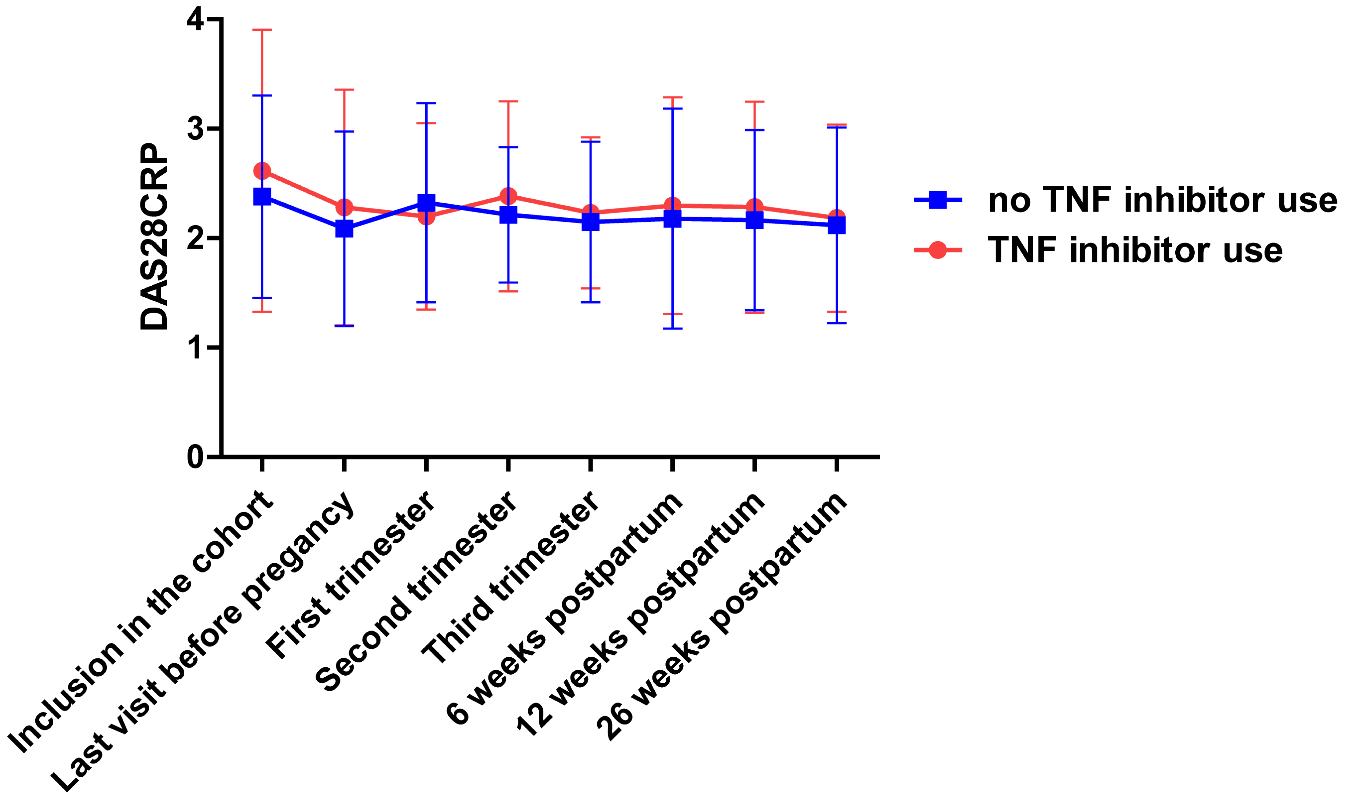

Supplementary figure 1: Graph showing DAS28CRP (mean, SD) scores over time in the PreCARA cohort (T2T-cohort) stratified for TNF inhibitor use during pregnancy (any use during pregnancy). The x-axis displays specific time-points before, during and after pregnancy and the y-axis represents mean (SD) disease activity. Disease activity over time did not differ between patients who used a TNF inhibitor during pregnancy (any use during pregnancy) and patients who did not (p = 0.14).
